# Properties of PTFE tape as a semipermeable membrane in fluorous reactions

**DOI:** 10.3762/bjoc.11.110

**Published:** 2015-06-09

**Authors:** Brendon A Parsons, Olivia Lin Smith, Myeong Chae, Veljko Dragojlovic

**Affiliations:** 1 Wilkes Honors College of Florida Atlantic University, 5353 Parkside Drive, Jupiter, FL 33458, USA; 2 Department of Chemistry, University of Washington, Box 351700, Seattle, WA 98195-1700, USA

**Keywords:** perfluorinated solvents, phase screen, PTFE, semipermeable membrane, synthesis design

## Abstract

In a PTFE tape phase-vanishing reaction (PV-PTFE), a delivery tube sealed with PTFE tape is inserted into a vessel which contains the substrate. The reagent diffuses across the PTFE tape barrier into the reaction vessel. PTFE co-polymer films have been found to exhibit selective permeability towards organic compounds, which was affected by the presence of solvents. In this study, we attempted to establish general trends of permeability of PTFE tape to different compounds and to better describe the process of solvent transport in PV-PTFE bromination reactions. Though PTFE tape has been reported as impermeable to some compounds, such as dimethyl phthalate, solvent adsorption to the tape altered its permeability and allowed diffusion through channels of solvent within the PTFE tape. In this case, the solvent-filled pores of the PTFE tape are chemically more akin to the adsorbed solvent rather than to the PTFE fluorous structure. The solvent uptake effect, which was frequently observed in the course of PV-PTFE reactions, can be related to the surface tension of the solvent and the polarity of the solvent relative to the reagent. The lack of pores in bulk PTFE prevents solvents from altering its permeability and, therefore, bulk PTFE is impermeable to most solvents and reagents. However, bromine, which is soluble in liquid fluorous media, diffused through the bulk PTFE. A better understanding of the PTFE phase barrier will make it possible to further optimize the PV-PTFE reaction design.

## Introduction

Fluorous reactions are a relatively new addition to the portfolio of synthetic methods [1–5]. In a fluorous triphasic reaction, introduced by Curran in 2001, a fluorous phase screen was used to simultaneously carry out the reaction and separate the products [6–7]. Soon after, Ryu and Curran reported the fluorous phase-vanishing (PV) method, which unlike the triphasic fluorous reaction, does not require the use of fluorous reactants [8]. In the course of the reaction, one phase vanishes, hence the name. An important requirement of PV reactions is that, in order to ensure an effective reaction, the rate of passive diffusion of the reagent through the fluorous phase to the substrate layer must be significantly greater than the diffusion of the substrate to the reagent layer [9]. Ryu and Curran’s work was followed by numerous improved and modified designs including a quadraphasic/tetraphasic PV reaction [10], a photochemical PV reaction [11–12], a stacked phase-vanishing reaction [13], a PV reaction on neat substrates [11,14–16] and, most recently, a Grignard reaction [17]. Jana and Verkade reported a variation of the PV method that allows the use of lighter reagents without the need for a U-tube by dissolving one of the reagents in a heavy solvent [18]. Curran and Werner established that such a reaction operates by an extractive mechanism, rather than the passive diffusion-controlled reagent transport observed in typical phase-vanishing techniques [19]. Curran followed up with a study of miscibilities and solubilities of fluorous/organic phases [20].

**Figure 1:**
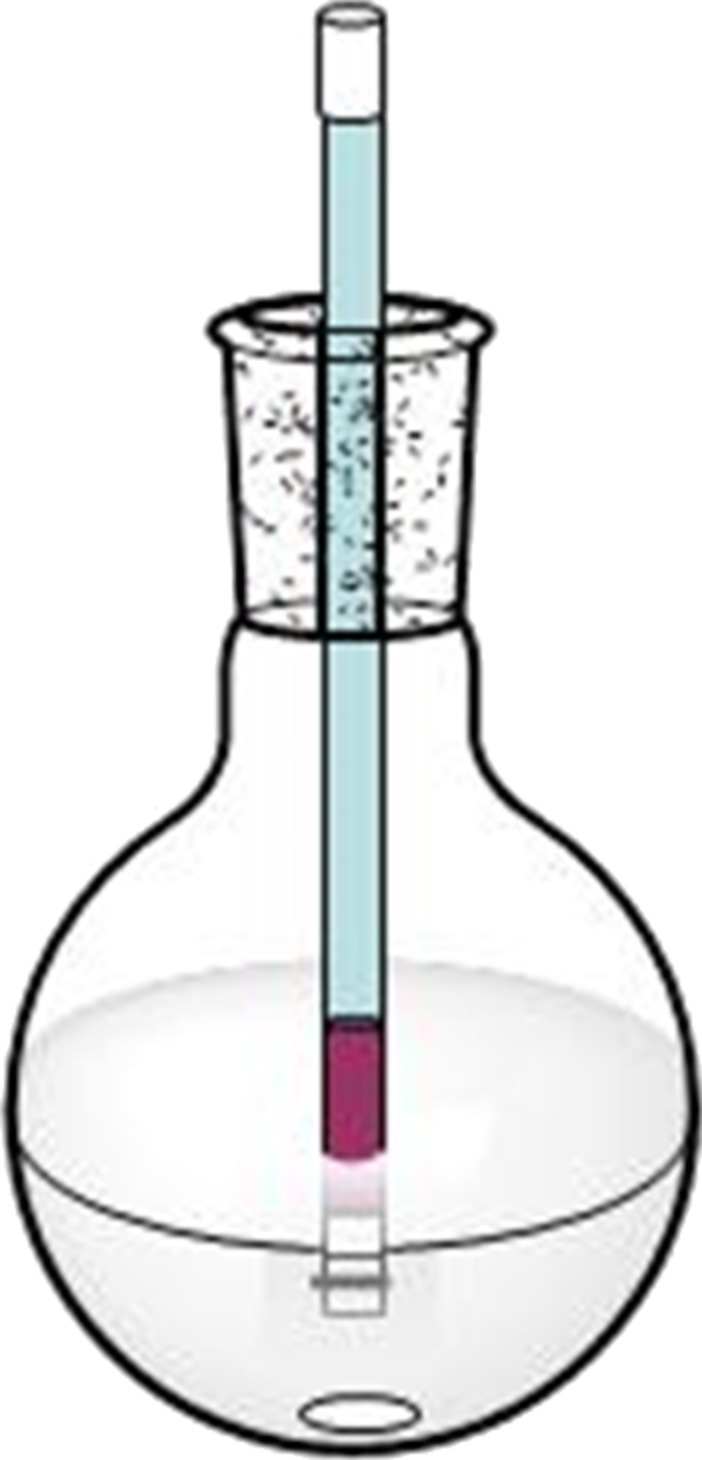
PV-PTFE reaction design.

Following the improvements that resulted from use of heavier polymeric liquid perfluoro compounds reported by Ryu et al. [10], we introduced PTFE (polytetrafluoroethylene, Teflon) tape as a phase screen [21–23]. In a PTFE tape phase-vanishing reaction (PV-PTFE), a delivery tube containing the reagent is sealed on both ends with PTFE tape. The PTFE tape covering the top of the delivery tube prevents the loss of the reagent over the course of the reaction. The delivery tube is then inserted into a vessel which contains substrate, either neat or dissolved in a solvent. The reagent diffuses across the PTFE tape barrier into the outer vessel (Figure 1). The reaction generally takes place in the vessel, although in some instances due to the diffusion of the substrate through the PTFE tape the product also formed in the delivery tube. The PV-PTFE method is inexpensive, simple to use and more environmentally friendly. Furthermore, the delivery of the reagent can easily be stopped and the reaction products are easy to recover. PV-PTFE reactions have no limitations due to the densities of the reagents or substrates and they can be used in sequential or tandem reactions, or reactions under reflux [21–23]. We also utilized alternative PTFE-covered vials to simplify reagent handling [24].

The PV-PTFE method can be made more efficient by better understanding the phase barrier and its role in phase-vanishing reactions. Other PTFE films have been found to exhibit selective permeability towards organic compounds, and this permeability is affected by the presence of solvents. Weber and coworkers have reported such an investigation into the properties of Teflon AF (poly(2,2-bis(trifluoromethyl)-4,5-difluoro-1,3-dioxide-*co*-tetrafluoroethylene), a co-polymer with 13% PTFE content [25–26]. A Teflon AF 2400 tubing was also used in a reaction to deliver a gaseous reactant by Ley [27]. Weber carried out a quantitative study on Teflon AF films that were carefully cast and well-characterized [25–26]. As we used commercially available PTFE tape for which tolerances are considerably higher, drawing general reproducible conclusions may be more difficult.

PTFE has found use as both a catalyst carrier and a means of catalyst recovery by Gladysz [28] and as a support when taking IR spectra [29]. The most common everyday laboratory use of PTFE is as a protective coating of magnetic stirring bars. Bulk PTFE is chemically identical to PTFE tape. However, Dihn and Gladysz reported that it behaves very differently in reactions involving fluorous catalyst recovery, and that study into the morphology of PTFE was needed [30]. To better understand how bulk and tape PTFE perform, we investigated their properties with respect to some common reagents and reaction conditions. In this paper, which was designed as a preliminary study, we attempted to establish general trends of the permeability of PTFE to different compounds, to investigate whether organic solvents alter it, and to better describe the process of solvent transport in PV-PTFE bromination reactions. We also compared PTFE tape to bulk PTFE, which is typically encountered as an inert protective coating, such as on laboratory stir bars.

## Results and Discussion

### Solvent transport in the course of a simple PV-PTFE diffusion of bromine into solvent

**Figure 2:**
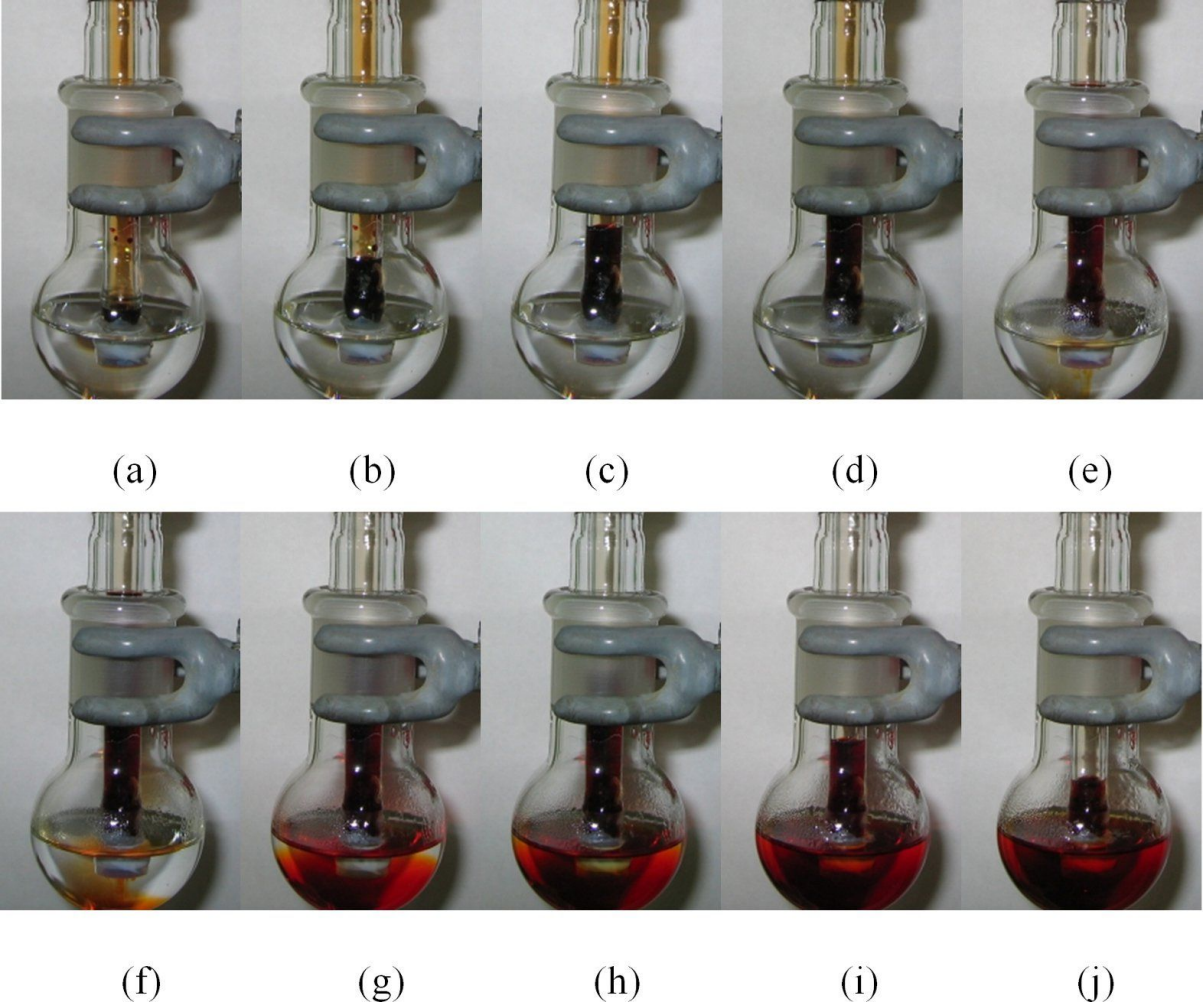
Solvent uptake in the delivery of bromine into dichloromethane (a) 0 min, (b) 0.50 min, (c) 0.83 min, (d) 1.50 min, (e) 3.50 min, (f) 4.17 min, (g) 7.18 min, (h) 10.18 min, (i) 17.18 min, (j) 23.57 min. Note that a part of the delivery tube was obscured by a ground glass joint. The solvent column reached the maximum height ≈3.5–4 min (just above the top of the ground glass joint) and at that point, the delivery of bromine to the flask began.

To better understand the solvent uptake phenomenon observed in PV-PTFE reactions with dichloromethane as a solvent [21] we investigated the addition of bromine through a PTFE-sealed delivery tube to a solvent that did not contain any substrate. In the delivery of bromine into dichloromethane, one may expect that the denser reagent in the delivery tube would simply diffuse downward into the less dense solvent. Indeed, when a delivery tube of bromine was placed in an empty flask, bromine vapors were observed diffusing through the PTFE tape and descending to the bottom of the flask [22]. However, when the tip of the delivery tube was immersed in dichloromethane, initially the solvent in the flask was quickly drawn up into the delivery tube. At this point, relatively little bromine had diffused into the solvent in the flask (Figure 2). Next, after the column height of the bromine-solvent mixture reached a maximum, it slowly decreased. During this phase, most of the bromine was delivered to the flask.

**Figure 3:**
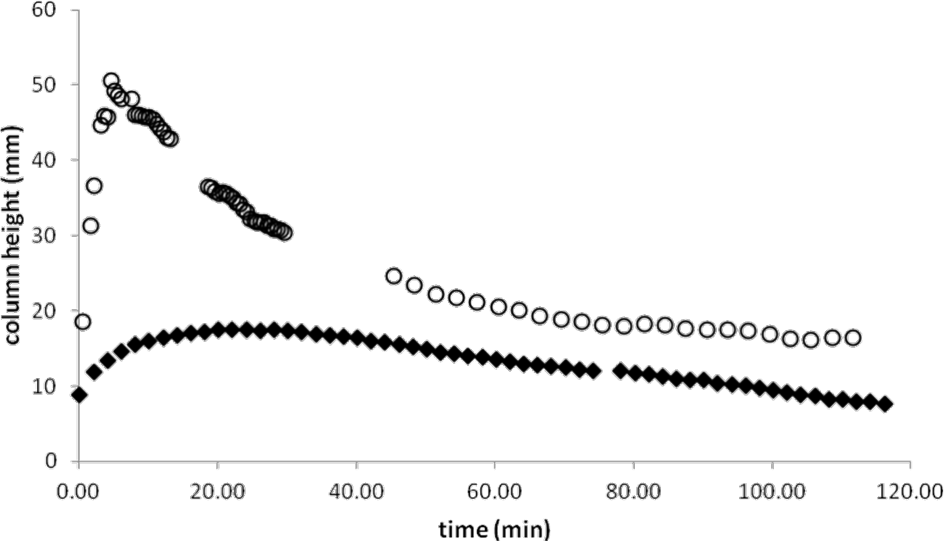
Solvent column heights of bromine delivery into dichloromethane (○) and ethyl acetate. (♦).

The solvent uptake also occurs with other organic solvents. We repeated the experiment with ethyl acetate and hexanes. Ethyl acetate exhibited solvent transport similar to that observed with dichloromethane. However, the column height reached a lower maximum and both the uptake and drawdown processes were slower (Figure 3). Unfortunately, we were unable to obtain good results for hexanes, as the uptake of the solvent was rapid and the column of solvent overflowed the delivery tube.

**Figure 4:**
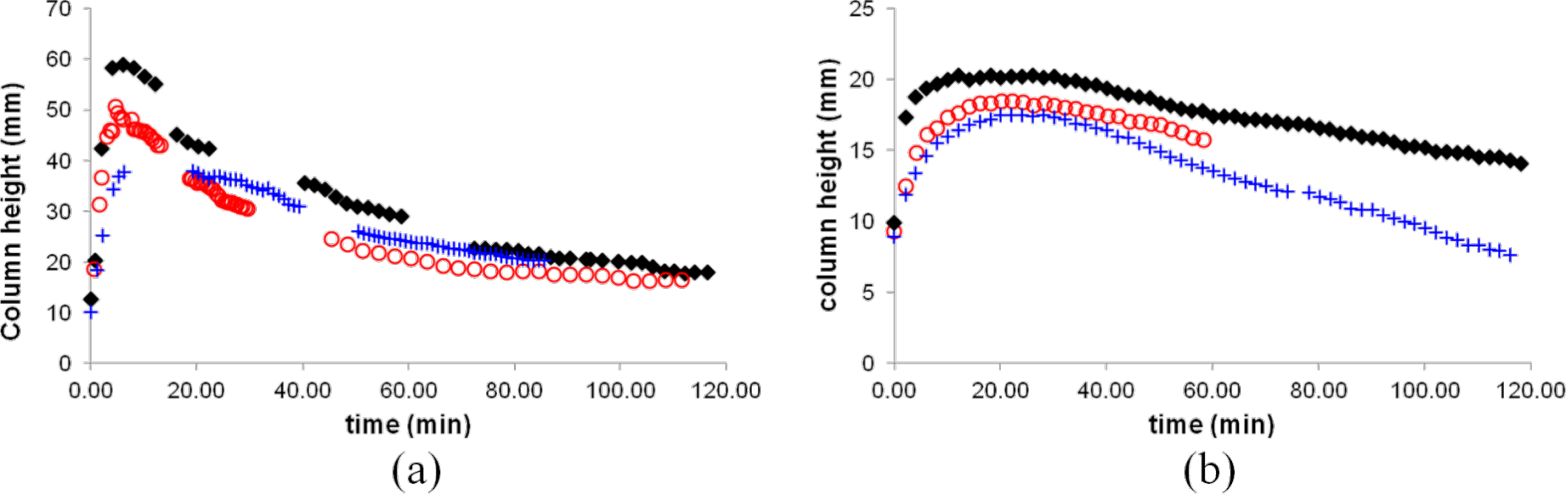
Reproducibility of bromine delivery into a) dichloromethane and b) ethyl acetate. In each case three different trials are shown.

PTFE tape is easily stretched, which might cause a change in the size of pores. This may explain why in different trials we obtained slightly different results (Figure 4). Variations were considerably greater in trials involving dichloromethane.

### Solvent transport in the course of PV-PTFE bromination of cyclohexene

**Figure 5:**
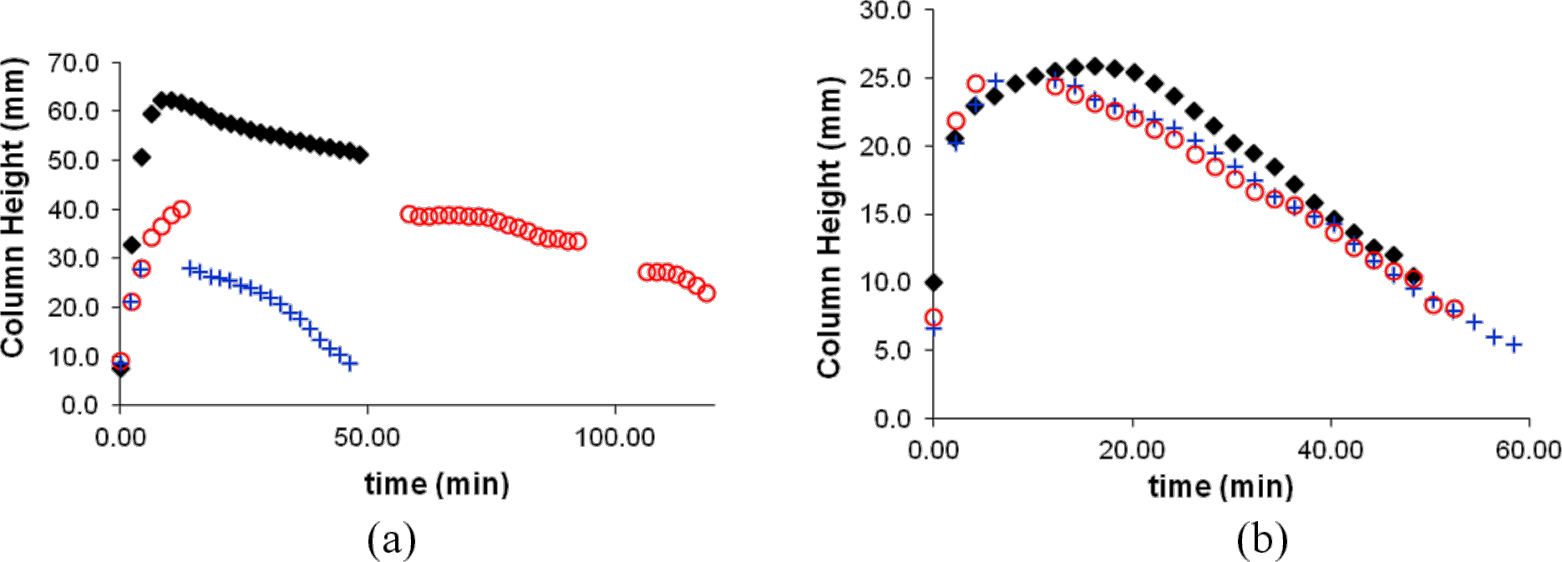
Height of the solvent column in the course of the bromination of cyclohexene in (a) dichloromethane and (b) ethyl acetate. In each case three different trials are shown.

To examine the solvent transport effect in the course of a reaction, we performed the delivery of bromine to an equimolar amount of cyclohexene dissolved in a solvent. Due to the previously described problem with hexanes overflowing the delivery tube, we investigated only reactions in dichloromethane and ethyl acetate. Figure 5 represents the heights of the liquid columns in the delivery tubes over the course of the reaction. Again, there are gaps present due to the ground glass joints preventing the observation of the level of the bromine–solvent mixture in the delivery tube. As with the previous experiments, the trials with dichloromethane were less reproducible in terms of the maximum column height reached and the amount of time it took for the solvent drawdown to complete. Trials involving ethyl acetate were more reproducible. Most of the variation was in the time it took for the solvent column to reach its maximum. The amount of solvent that entered the delivery tube and the time until the delivery tube was drawn down were relatively consistent.

**Figure 6:**
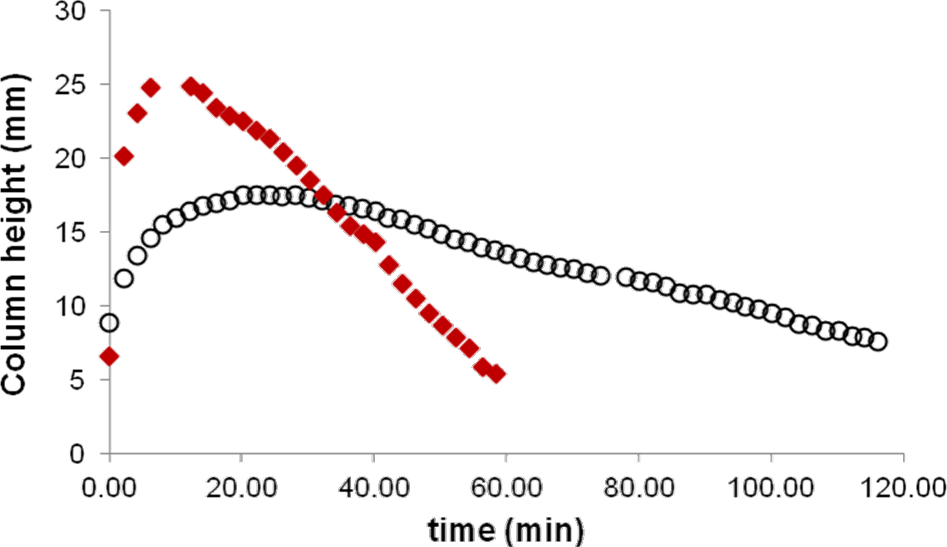
Height of the solvent column in the course of the bromination of cyclohexene in ethyl acetate (♦) and the delivery of bromine to neat ethyl acetate (○).

Compared to the simple delivery of bromine into ethyl acetate, the bromination of cyclohexene in ethyl acetate resulted in a bromine–solvent column which rose faster and reached a higher maximum (Figure 6). Additionally, in the presence of a substrate, the drawdown process was faster.

Therefore, we have found that the solvent uptake effect reported earlier [21] also occurs without a substrate. We have also found that the process of solvent transport varies with the solvent used. The implication for the PV-PTFE method is that even once a solvent is chosen, the rate at which the reagent is delivered may still vary between individual trials. A possible solution to this problem is to place bromine, or the reagent in general, dissolved in a suitable amount of solvent, into the delivery tube rather than using a neat reagent.

**Figure 7:**
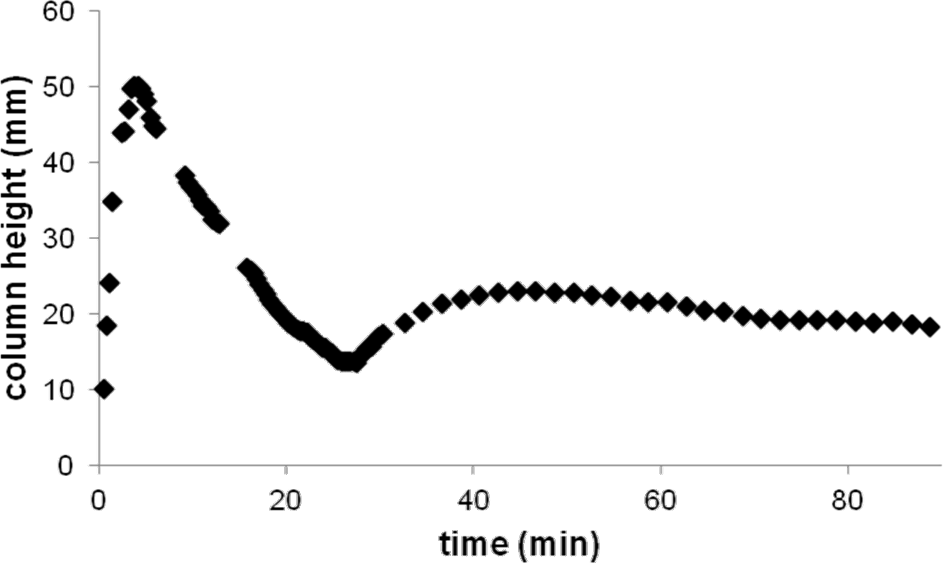
Solvent uptake when the delivery tube is inserted to a shallow depth. The solvent uptake stopped once the level of the solution reached the bottom of the delivery tube. After some solution flowed from the tube back into the reaction vessel and the liquid level reached the tube, the uptake of the solvent resumed.

The solvent uptake process can be controlled to an extent by varying the depth to which the delivery tube is immersed in the solution. If the tube is immersed to a greater depth, the solvent can be drawn into the tube until equilibrium is reached. However, if the delivery tube is immersed into the solution to a shallow depth, the uptake into the delivery tube draws down the level of solvent in the reaction vessel, and the uptake stops once the level of the solution in the reaction vessel is below the bottom of the delivery tube. Then, the solution flows from the delivery tube back into the reaction vessel until the liquid level reaches the delivery tube again, at which point the uptake of the solvent resumes (Figure 7).

**Scheme 1:**
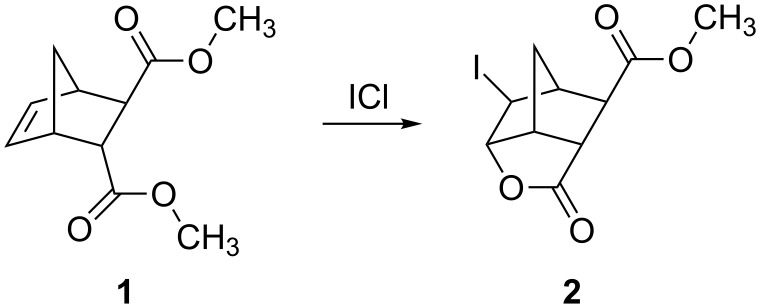
Iodolactonization of unsaturated diester **1** with iodine monochloride in dichlormethane.

**Figure 8:**
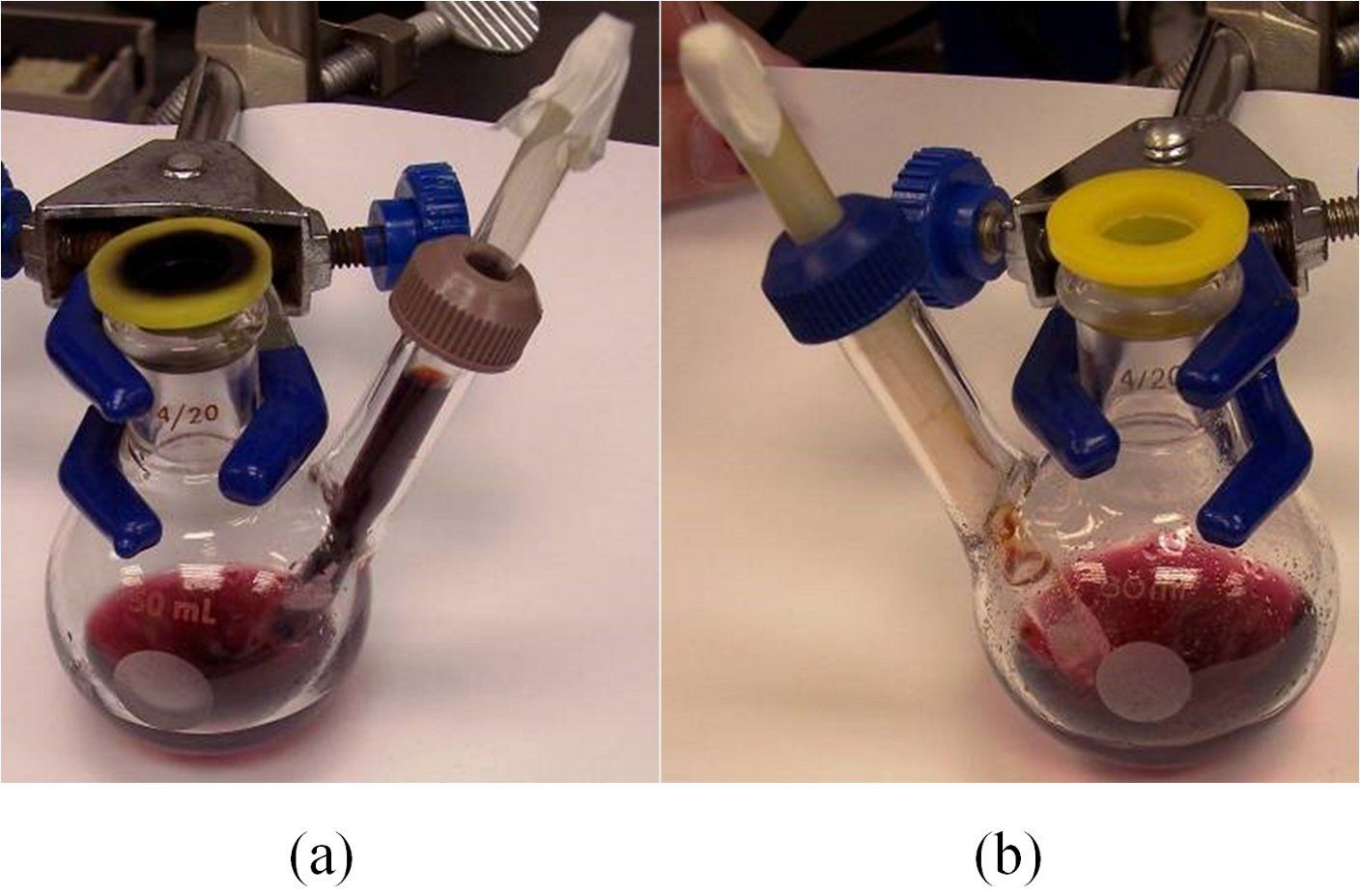
(a) The delivery tube is immersed into the solution and there is a considerable solvent uptake. (b) The delivery tube is just touching the surface of the solution and there was almost no solvent uptake.

By having the delivery tube just touch the surface of the solution in the reaction vessel, one can almost completely eliminate the solvent uptake as demonstrated by the PV-PTFE iodolactonization of the unsaturated diester **1** with iodine monochloride in dichlormethane as a solvent (Scheme 1 and Figure 8).

### Solvent-assisted transport through PTFE tape

**Figure 9:**
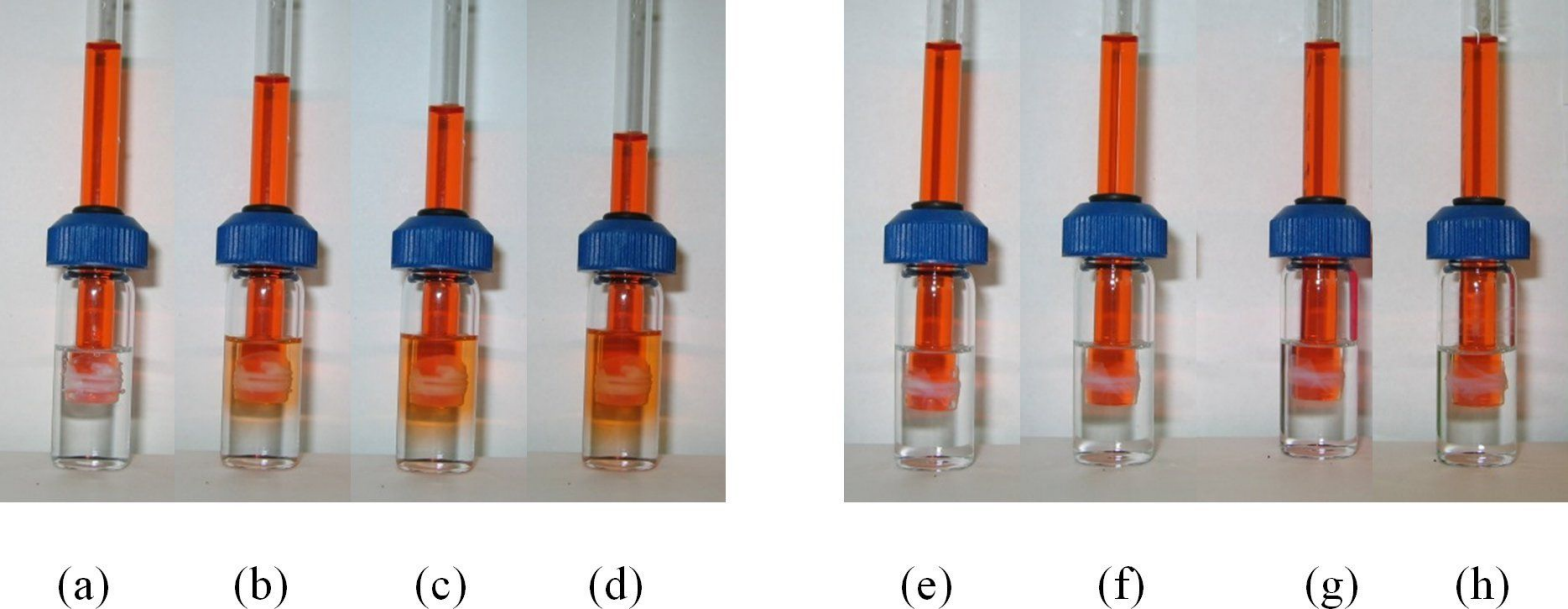
Transport of dyed dimethyl phthalate in dichloromethane after (a) 0 h, (b) 1 h, (c) 2 h, (d) 3 h and dyed dimethyl phthalate in hexanes after (e) 0 h, (f) 1 h, (g) 2 h, (h) 3 h.

In the earlier work we reported that PTFE tape is impermeable to dimethyl phthalate [21]. However, we suspected that solvent effects may alter the permeability of PTFE tape even when it is otherwise impermeable to a compound. In order to visualize the flow of solvents, a small amount of methyl red was dissolved in dimethyl phthalate. When a delivery tube containing dyed dimethyl phthalate was suspended in an empty beaker, the column height did not change at all, even after three days. However, when the same delivery tube was immersed in dichloromethane, the dyed dimethyl phthalate was able to flow out of the delivery tube almost immediately (Figure 9a–d). When a delivery tube of dyed dimethyl phthalate was similarly immersed in hexanes, the phthalate did not cross the PTFE tape barrier (Figure 9e–h).

**Figure 10:**
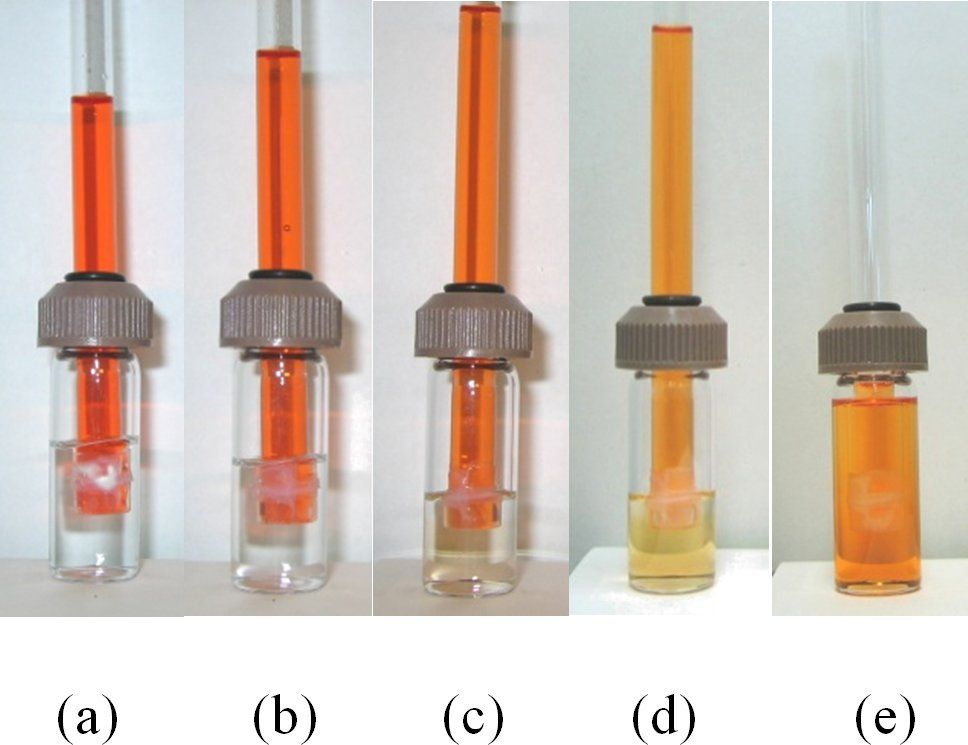
Transport of dyed dimethyl phthalate in ethyl acetate after (a) 0 h, (b) 0.17 h, (c) 1 h, (d) 3 h, (e) 30 h.

The delivery tube immersed in ethyl acetate exhibited a solvent-assisted phthalate delivery that was somewhat different compared to one observed with dichloromethane. The column height of the delivery tube increased indicating that, while dyed phthalate was exiting the delivery tube, ethyl acetate was also drawn into it. At some point (≈1–2 h into the process), the column height reached a maximum, which was followed by a relatively slow transport of the contents of the delivery tube into the outer vial (Figure 10).

As with the experiments on the transport of bromine, it is apparent that the nature of the solvent plays a major role in the transport of dimethyl phthalate across the PTFE tape barrier. PTFE tape is characterized at the microscopic level as a highly-porous solid. These pores are estimated from scanning electron micrographs to be about 0.5 μm in size, though the pores may change in size and number if the tape is stretched [29,31]. Weber and coworkers found that solvent-saturated Teflon AF films are not truly fluorous and that the solvent creates a supported-liquid membrane which drastically changes the diffusion properties of solutes through the film [25–26]. Based on our experimental observations, the structure of PTFE tape, and results of investigation of Teflon AF reported by Weber, we propose that compatible solvents adsorb to the PTFE tape and fill in the voids in the tape. The solvent-filled pores of the PTFE tape then are more chemically akin to the solvent rather than the PTFE tape. Therefore, compounds that do not diffuse through PTFE tape, such as dimethyl phthalate, will be able to diffuse through in the presence of a suitable solvent. To be suitable as a transport medium, a solvent must meet two criteria.

First, the solvent must be capable of adsorbing to the PTFE tape. This characteristic is visibly displayed by “wetting” of the PTFE tape by the solvent and is related to the contact angle that results between the solvent and the solid. PTFE tape is wetted spontaneously by any solvent which has a critical surface tension (γ_C_) of less than 27 × 10^−3^ N m^−1^ [32]. In the course of our experiments, we observed that dichloromethane (26.50 × 10^−3^ N m^−1^) [33], hexanes (18.43 × 10^−3^ N m^−1^) [33], and ethyl acetate (23.9 × 10^−3^ N m^−1^) [34] all wet PTFE tape. Acetonitrile (29.29 × 10^−3^ N m^−1^) [35], water (72.80 × 10^−3^ N m^−1^) [33], and dimethyl phthalate (41.85 × 10^−3^ N m^−1^) [36] did not wet PTFE tape.

Second, the compound of interest must be fairly soluble in the solvent which has filled the void space in the PTFE tape. Dimethyl phthalate is insoluble in solvents which are non-polar (aliphatic hydrocarbons such as hexanes) or highly-polar (water), but dissolves well in medium-polarity solvents such as dichloromethane and ethyl acetate [37]. Even though all the solvents used in this experiment adsorbed on the PTFE tape, dimethyl phthalate crossed the PTFE tape barrier only when the medium-polarity solvents (dichloromethane and ethyl acetate) were used. When either the non-polar hexanes or polar water (vide infra: chemiluminescence experiment) were employed, dimethyl phthalate did not diffuse through the tape-adsorbed solvent at a measurable rate.

### Selective reagent transport in PV-PTFE chemiluminescence

**Scheme 2:**
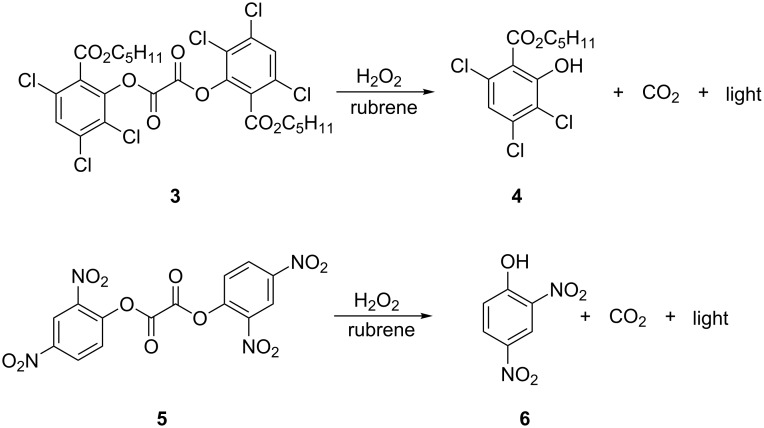
Chemiluminescence reaction of diaryl oxalate esters oxidized by hydrogen peroxide in the presence of rubrene.

To visualize the transport across a PTFE barrier, we designed a PV-PTFE chemiluminescence experiment as a qualitative test. The known chemiluminescence reaction of the diaryl oxalate esters oxidized by hydrogen peroxide in the presence of rubrene was investigated (Scheme 2) [38–40]. Two solutions were prepared. One solution contained a mixture of diaryl oxalate and rubrene in dimethyl phthalate. The second solution was hydrogen peroxide dissolved in either water, or a water–organic solvent mixture.

**Figure 11:**
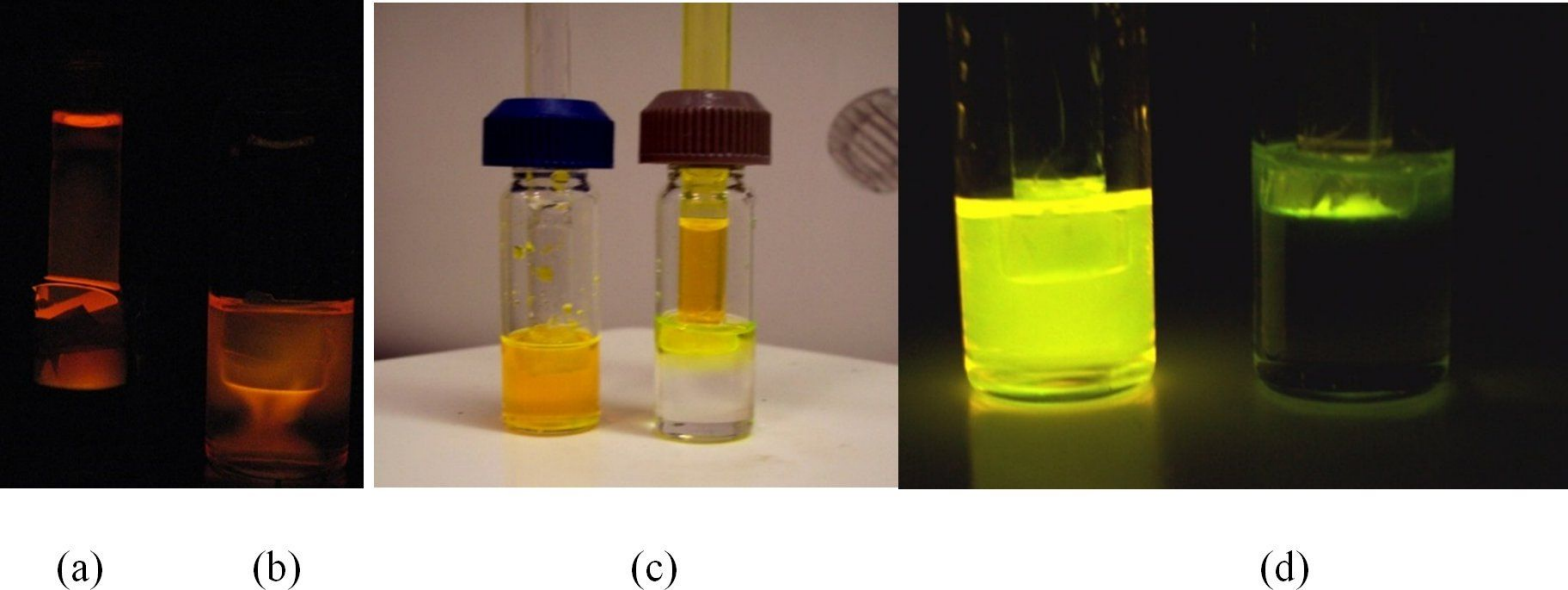
When the diaryl oxalate was oxidized by aqueous peroxide solution, chemiluminescence was observed only in the oxalate–rubrene solution, which was (a) in the delivery tube in the experiment on the left and (b) in the vial in the experiment on the right. (c) The experimental set up with the lights on and (d) lights off. The orange solution contains an oxalate/rubrene mixture in dimethyl phthalate and the clear solution is 10% aqueous H_2_O_2_.

The solutions in the vial and delivery tube were alternated to ensure that the direction of diffusion was not gravity or density-dependent. We observed that the chemiluminescence reaction was directional. Only the oxalate–rubrene solution exhibited luminescence during the reaction, regardless of whether it was in the delivery tube or the vial (Figure 11). The peroxide solution remained dark.

**Figure 12:**
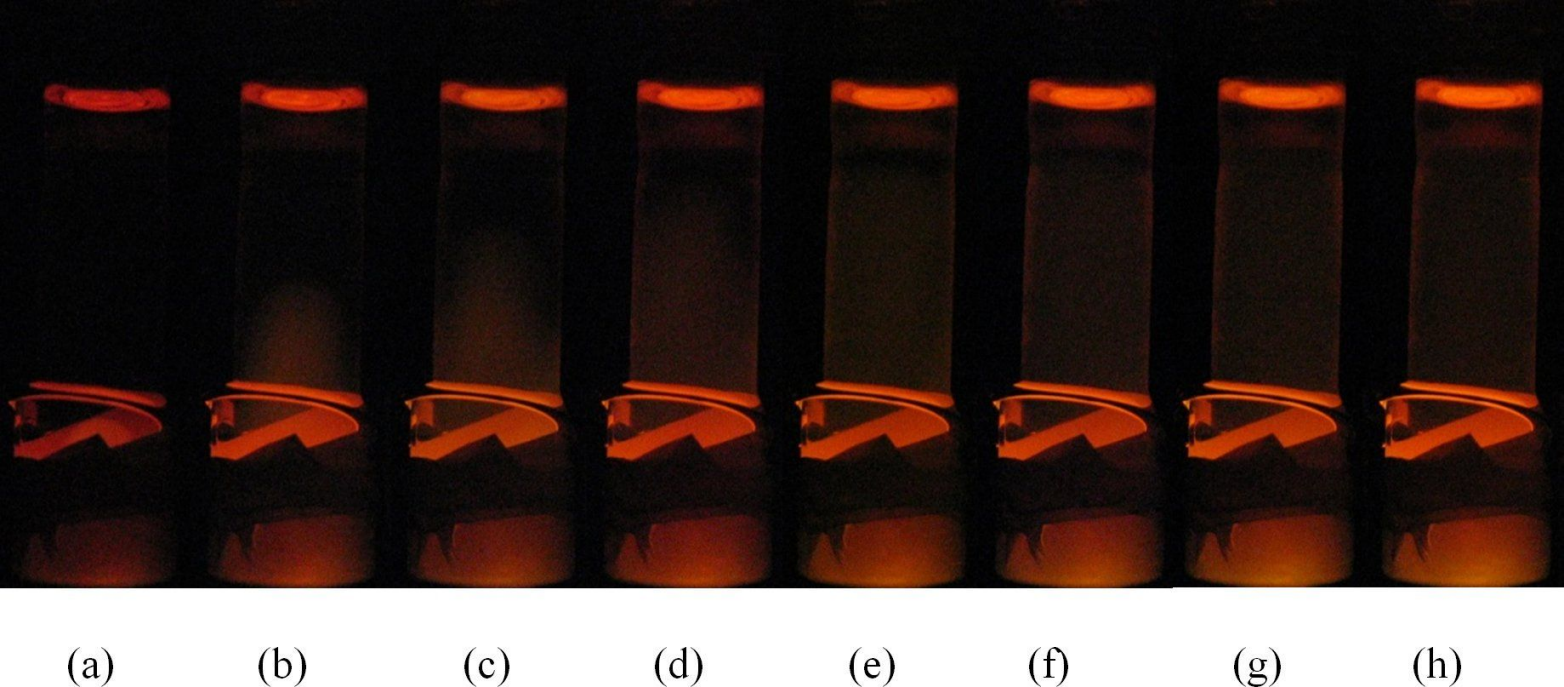
Progression of PV-PTFE chemiluminescence with aqueous peroxide solution in the vial after (a) 10 min, (b) 30 min, (c) 50 min, (d) 70 min, (e) 90 min, (f) 110 min, (g) 130 min, (h) 180 min.

The reactions with the aqueous peroxide solution proceeded with no change in the column height of the oxalate–rubrene solution in the delivery tube (Figure 12), even over an extended period of time. This indicates that solvent transport was not necessarily a requirement for the delivery of the H_2_O_2_ reagent. From the directionality of the PV-PTFE chemiluminescence reaction and lack of change in solvent volume in the delivery tube, we conclude that PTFE tape is permeable to small H_2_O_2_ molecules but not to the considerably larger oxalate or rubrene molecules.

**Figure 13:**
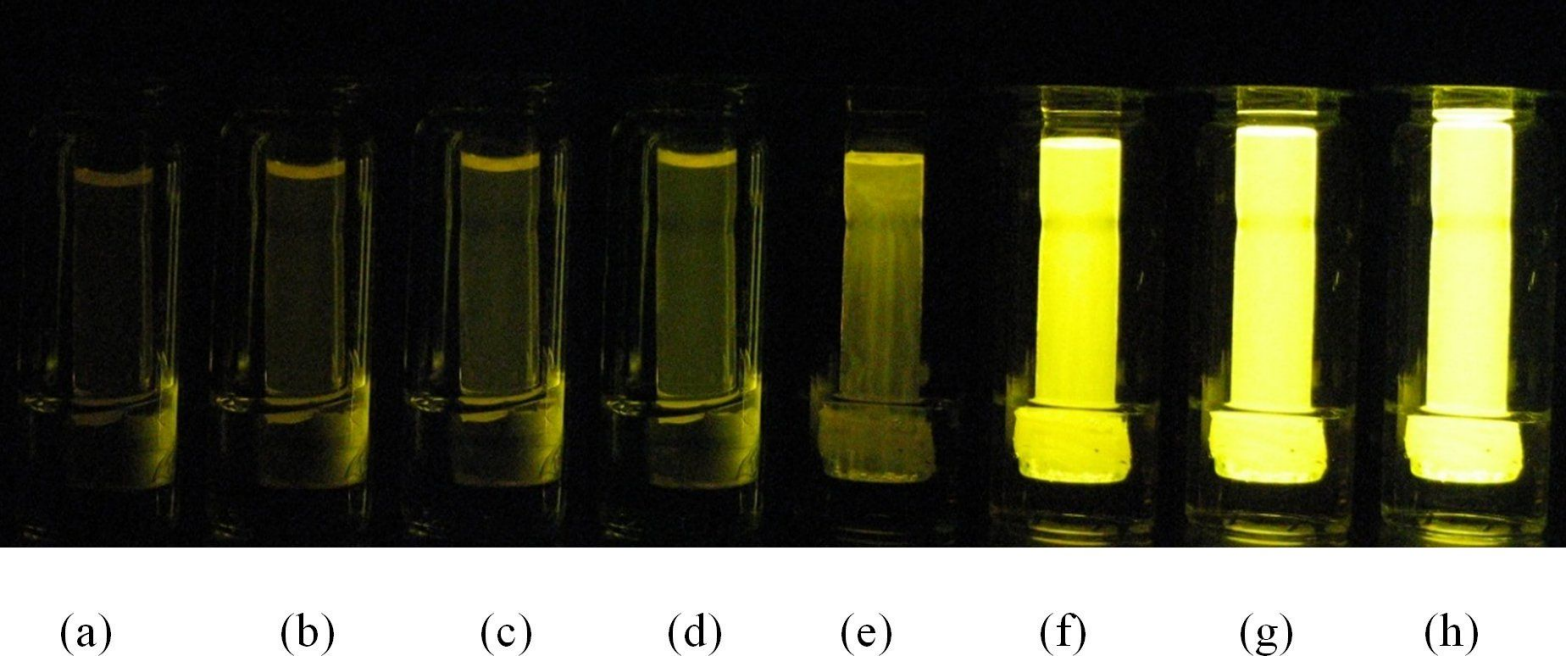
Progression of PV-PTFE chemiluminescence with acetonitrile–aqueous peroxide solution in the vial after (a) 10 min, (b) 15 min, (c) 20 min, (d) 25 min and *tert*-butanol–aqueous peroxide solution in the vial after (e) 5 min, (f) 10 min, (g) 15 min, (h) 20 min.

In the reactions where hydrogen peroxide was dissolved in acetonitrile/water (2:1) or *tert*-butanol/water (2:1) mixtures, the column height changed during the reaction. This effect was similar to the phenomenon observed in the course of PV-PTFE brominations and solvent-assisted transport of dimethyl phthalate. When the peroxide–solvent solution was in the delivery tube, the column height decreased over the course of the reaction; when the peroxide–solvent solution was in the vial, the column height of the oxalate–rubrene solution in the delivery tube increased (Figure 13). Thus, some acetonitrile and *tert*-butanol diffused through the PTFE barrier. Therefore, we observed that some solvents such as acetonitrile are able to diffuse through PTFE tape, even if though they do not wet the tape by adsorption [32].

**Figure 14:**
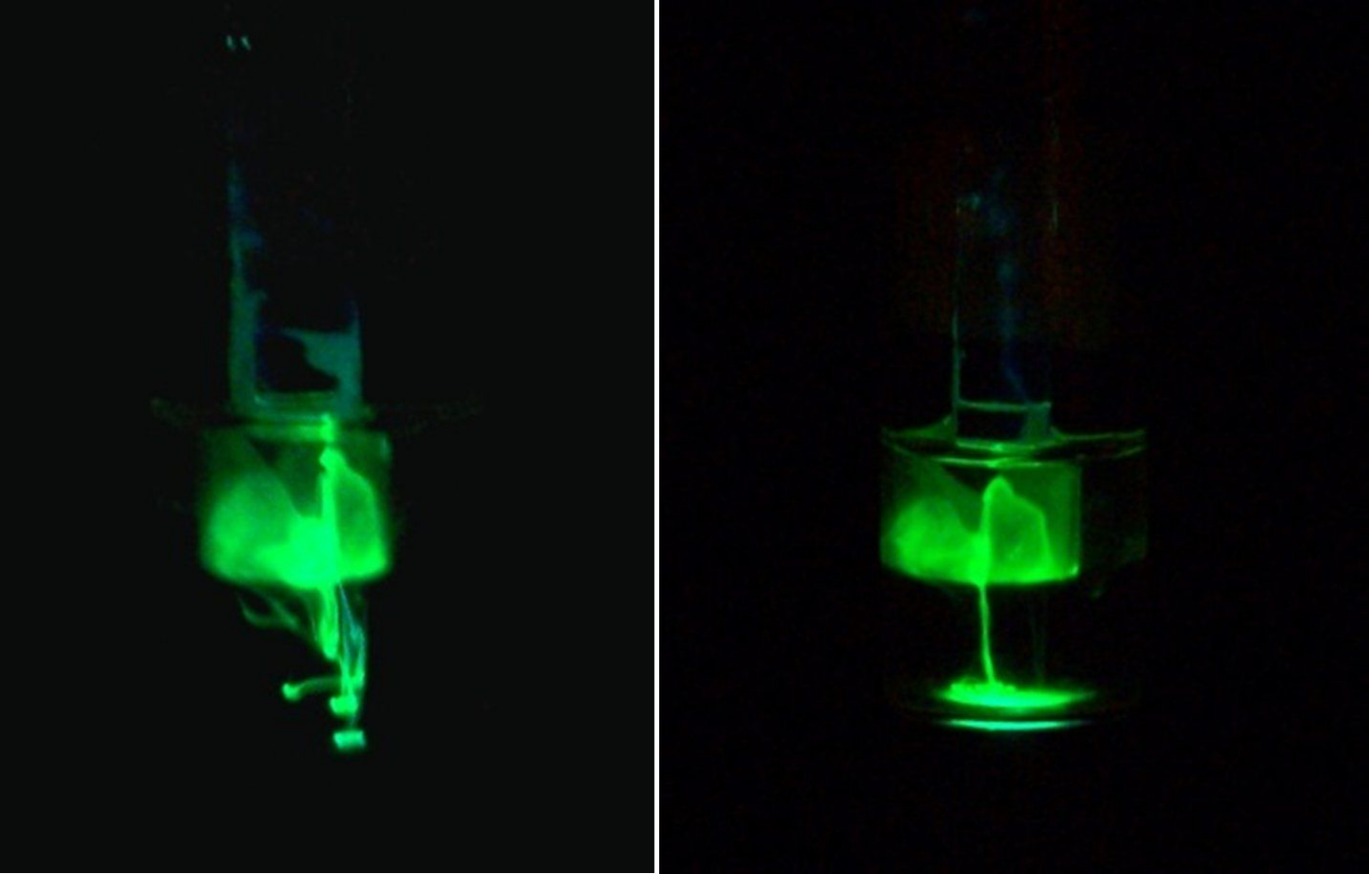
Diffusion of dimethyl phthalate assisted by *tert*-butanol through PTFE was visualized in a chemiluminescence reaction in which 9,10-bis(phenylethynyl)anthracene and an oxalate ester in dimethylphthalate diffused into a solution of hydrogen peroxide (10%) in a mixture of *tert*-butanol and water (2:1).

Finally, when peroxide in *tert*-butanol/water (2:1) was the oxidant and 9,10-bis(phenylethynyl)anthracene the acceptor, solvent-assisted diffusion of dimethyl phthalate was visualized (Figure 14). One can observe bright green streams of dense dimethyl phthalate solution as it diffuses through PTFE tape and pools at the bottom of the vial.

### Solvent-free diffusion of bromine through PTFE tape

**Figure 15:**
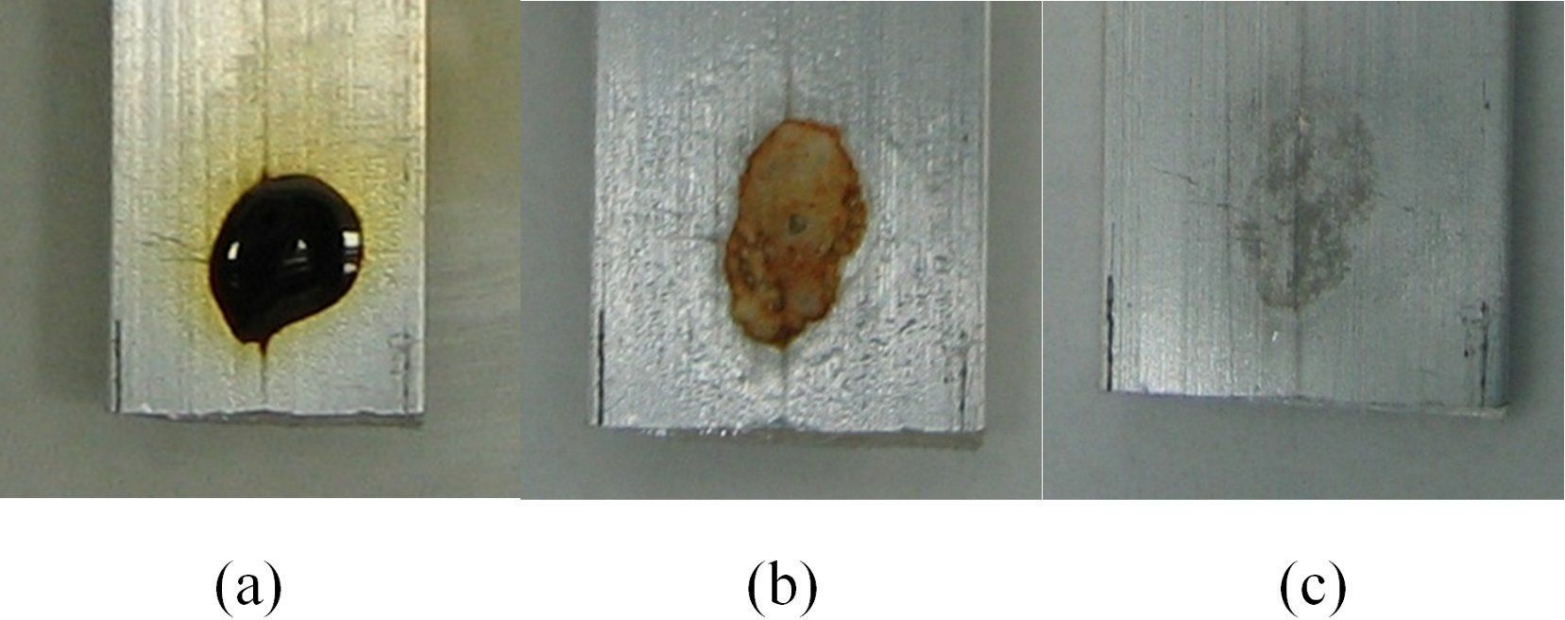
Corrosion of aluminum resulting from bromine applied directly to metal.

**Figure 16:**
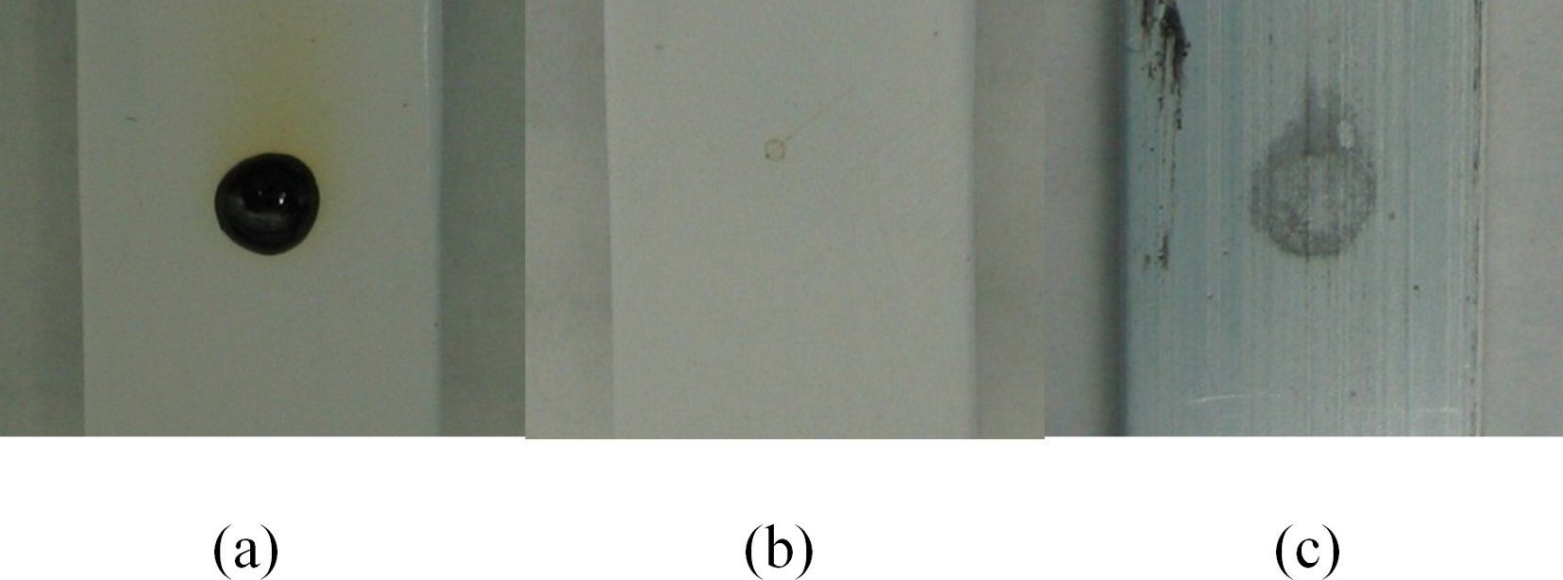
Discoloration of aluminum from bromine applied to PTFE tape on metal.

Solvent-free diffusion of bromine through PTFE tape was observed when a PV-PTFE bromination was carried out in the absence of a solvent on a neat substrate [22]. Furthermore, when a delivery tube of bromine was placed in an empty container, it was relatively quickly filled with bromine vapors. As an additional test of bromine diffusion through PTFE tape in the absence of a solvent, we utilized the reaction of bromine with aluminum, which discolors the metal. A single drop of bromine was placed on two sections of an aluminum bar; one section was exposed aluminum (Figure 15a), the other section was covered with a single layer of PTFE tape (Figure 16a). The bromine was allowed to completely evaporate (Figure 15b and Figure 16b), at which point the PTFE tape was removed. We found that the discoloration of the aluminum that was covered with PTFE tape was consistent with the discoloration of the bare aluminum (Figure 15c and Figure 16c). Therefore, bromine readily and at a high rate diffused through PTFE tape in the absence of a solvent.

### Comparison of PTFE tape to bulk PTFE

Dihn and Gladysz reported that in the course of a recovery of fluorous catalysts, the catalysts attached to the PTFE tape, but not the PTFE-coated stir bar that was used in the reaction [30]. This difference in properties may be due to the microscopic structural differences between PTFE tape and the bulk PTFE used in inert coating applications. Unlike the porous structure of PTFE tape [29], bulk PTFE is a solid matrix with little in the way of surface irregularities [41]. Conversely, PTFE tape is characterized at the microscopic level as a highly-porous solid. As mentioned earlier, the pores are estimated from scanning electron micrographs to be about 0.5 μm in size and they may change in size and number if the tape is stretched [29,31]. The non-porous nature of bulk PTFE precludes the adsorption of other materials. This may be the reason why solvents do not spontaneously wet bulk PTFE as they do with PTFE tape. However, we predicted that since bromine is somewhat soluble in liquid fluorous media [19], it may exhibit similar solubility in solid PTFE and may be capable of diffusing through a bulk PTFE barrier.

**Figure 17:**
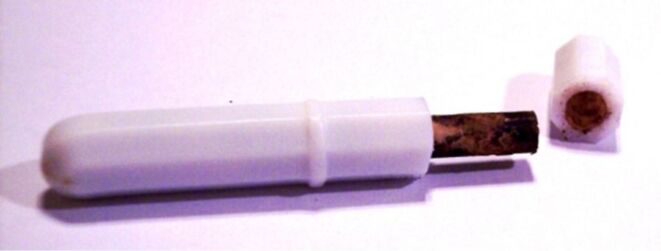
After stirring bars were cut open, some iron bars were found to be corroded.

**Figure 18:**
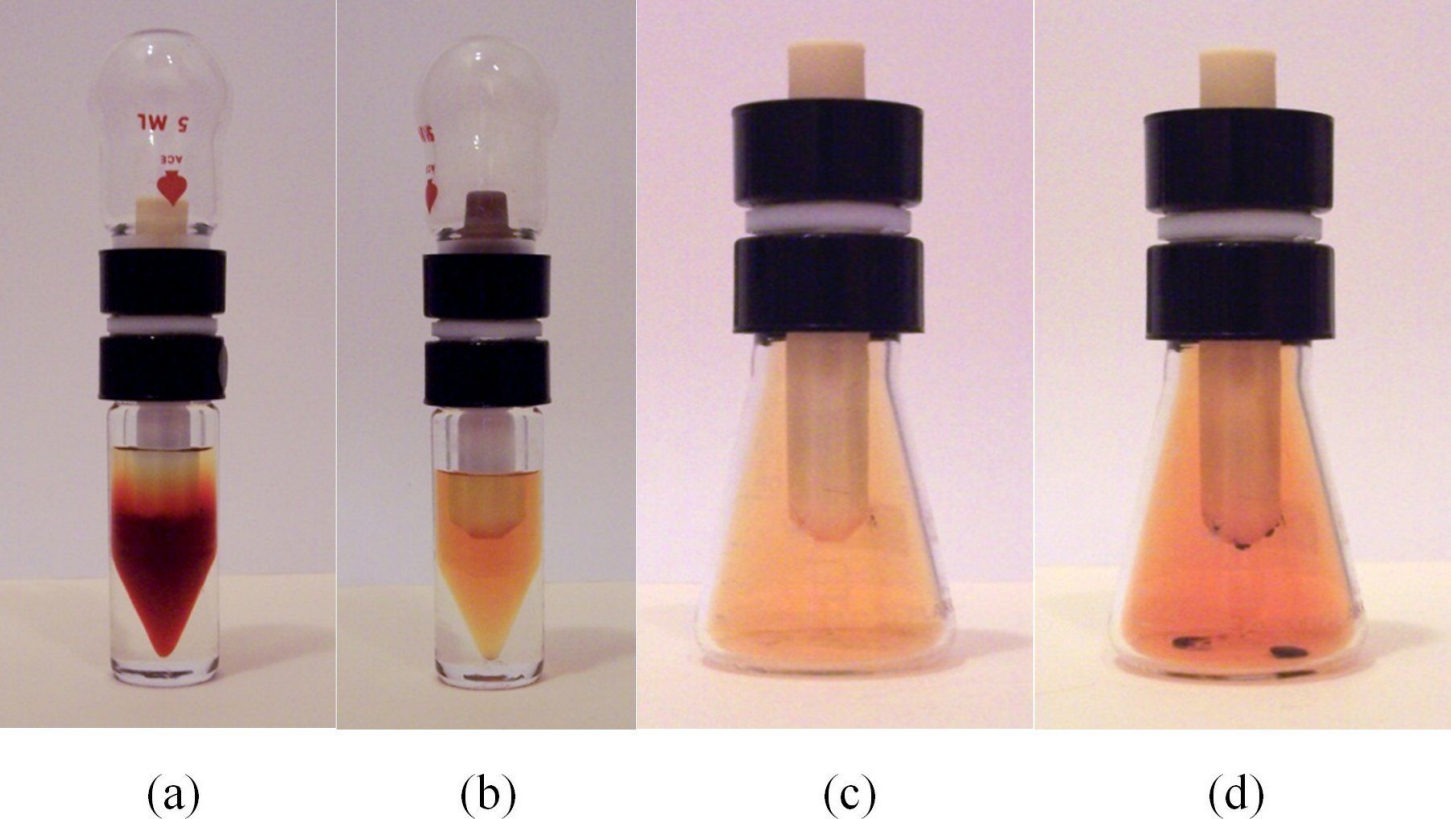
(a) Diffusion of bromine through a bulk PTFE from stirring bar into dichloromethane after 2 h. (b) Diffusion of bromine through a bulk PTFE from a different stirring bar of the same size into dichloromethane after 14 h. (c) Diffusion of bromine through a bulk PTFE into an empty flask after 2 h. (d) Diffusion of bromine through a bulk PTFE into an empty flask after 12 h.

We prepared a bulk PTFE vessel by cutting apart a laboratory stirring bar and removing the iron magnet. After cutting stirring bars open, we found that some of the iron bars were corroded (Figure 17). While we cannot exclude a possibility that the manufacturer used corroded bars, a more likely explanation is that they underwent corrosion with use. PTFE shell was cleaned, filled with bromine, closed with a septum and immersed in a vial containing dichloromethane (Figure 18a and b). To ensure that any bromine vapors that may leak through the stopper or a connection of it to PTFE vessel did not pass into the dichloromethane-containing vial, we used a Wheaton connector as a vial stopper and secured the PTFE container in it. Thus, the stopper on the PTFE container was external to the vial and any leak at it would be released outside the vial. We found that bromine did diffuse through the shell and that there was considerable variation between different shells of the same size (Figure 18a and b). To confirm that this was not a solvent-induced effect, we also repeated the experiment in an empty sealed vial. Bromine vapors filled the vial (Figure 18c) and eventually liquid bromine was observed (Figure 18d). Thus, though PTFE tape is chemically identical to bulk PTFE, we see that its microscopic structure markedly alters its behavior.

**Figure 19:**
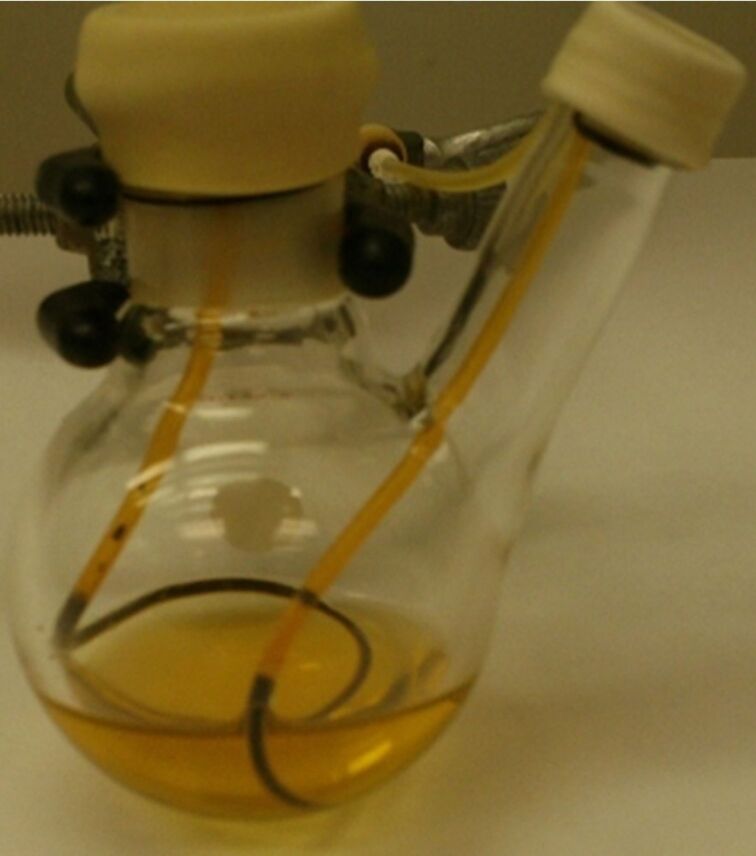
Diffusion of bromine through a PTFE tube.

We also tested the permeability of PTFE tubing in a set up that consisted of a two-neck flask and a PTFE tube. The PTFE tube entered the flask through two septa while both of its openings were sealed and were external to the flask (Figure 19). In this experiment, we again observed diffusion of the bromine across a bulk PTFE barrier.

**Figure 20:**
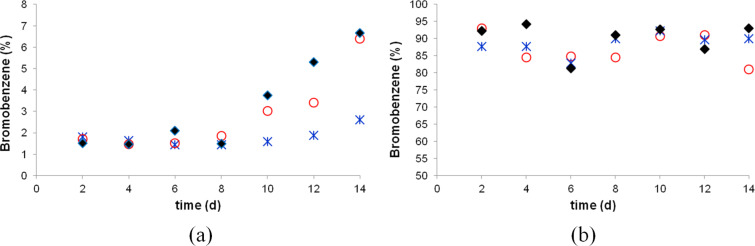
(a) The reaction of benzene and bromine in the absence of a stirring bar (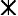), in the presence of a new stirring bar (○) and a used stirring bar (♦). (b) The reaction of benzene and bromine stirred by a cut stirring bar with the iron core exposed (three trials shown).

While bromine was observed to diffuse through the bulk PTFE, we did not observe any diffusion of solvents, including dichloromethane and ethyl acetate, when they were similarly contained in the PTFE container made from a stirring bar. Even though bromine diffuses through the bulk PTFE, as most solvents and substrates cannot diffuse through it, a reaction of the reagent with the stirring bar is not expected to have a direct effect on the reaction outcome. To test this hypothesis, we carried out a reaction of bromine with benzene. In the absence of a catalyst there is only a very slow reaction. However, in the presence of a Lewis acid catalyst there is a fast bromination reaction. Since iron reacts with bromine to produce iron(III) bromide, a stirring bar may conceivably catalyze the reaction provided that both bromine and benzene can diffuse through bulk PTFE. However, we found that with a stirring bar present, there was no acceleration of the reaction compared to the control (Figure 20a). There was a small increase in the conversion of benzene to bromobenzene starting after 8 days. For a comparison, a reaction was carried out with a cut stirring bar, so that its iron core was exposed, resulted in a very fast reaction and the formation of bromobenzene in a high yield (Figure 20b).

Magnetic stirring bars have been in use for a rather long time and, had they had any systematic effect on the reaction outcome, it likely would have been noticed a long ago. Nonetheless, one cannot exclude the possibility that there may be an effect due to diffusion through the PTFE coating in rare cases. Moreover, the diffusion of bromine and similar reagents through bulk PTFE may still affect the reaction outcome, as the stirring bar may consume some of the reagent. Finally, we would like to point out that we experienced some reproducibility problems (see Supporting Information File 1) and additional investigation of possible effects of PTFE-coated stirring bars on the reaction outcome may be warranted.

## Conclusion

To better understand how PTFE tape functions as a semipermeable membrane, we examined its behavior and compared it to bulk PTFE. We included both simple diffusion processes and chemical reactions to examine the effects of solvents and reagents on the permeability of the tape. We confirmed that when PV-PTFE reactions were carried out in a solvent, there was a solvent uptake effect. We found that the solvent uptake phenomenon varied according to the solvent, and that the effect is somewhat variable, especially with dichloromethane. By altering the solvent used in a PV-PTFE reaction, one may be able to control the rate of delivery of a reagent.

Though PTFE tape has been reported as impermeable to some compounds, such as dimethyl phthalate, we found that solvent adsorption to the tape alters its permeability. We believe that the porous microscopic structure of PTFE tape adsorbs solvents which fill in the void spaces. When the pores are filled with a suitable solvent, the compounds that cannot diffuse through PTFE tape instead dissolve in the adsorbed solvent, and then diffuse through channels of solvent within the PTFE tape. In this case, the PTFE tape is chemically more akin to the adsorbed solvent than its own fluorous structure. From the limited data available, it appears that this solvent effect can be related to the surface tension of the solvent and the polarity of the solvent relative to the reagent. If the surface tension is within the range necessary to adsorb to the pores of the PTFE tape, and the polarity is such that the reagent is soluble in the solvent, the otherwise impermeable reagent will be able to diffuse across the PTFE tape barrier.

Finally, the microscopic structural differences between PTFE tape and bulk PTFE are responsible for the observed differences in functionality. The lack of pores in bulk PTFE prevents solvents from altering its permeability. However, bromine diffused through the bulk PTFE. While our investigation was limited to bromine, other reagents soluble in liquid fluorous media should be tested with bulk PTFE.

## Experimental

### Solvent transport experiments

The 10 mL round bottom flask and delivery tube set-up (Figure 2) was placed next to a ruler within the camera frame. A Canon PowerShot S500 Digital Elph was set to take pictures on a time interval using Canon RemoteCapture software. The images were recorded in macro mode with auto-focus and flash enabled and ISO set to automatic. The maximum column height reached by the solution was quantified by measuring the number of vertical pixels in the column and correlating this number to pixel length of the ruler. The round bottom flask was filled with 5.0 mL of organic solvent. The delivery tube was inserted into the flask so that the lower tip of the delivery tube was immersed in the solvent. This process was repeated in triplicate with each solvent.

#### Solvent-free diffusion of bromine through PTFE tape

A single drop of bromine was placed on two sections of an aluminum bar; one section was exposed aluminum, the other section was wrapped with a single layer of PTFE tape. Bromine was allowed to completely evaporate, at which point the PTFE tape was removed. This section of the aluminum was then visually inspected for corrosion consistent with the section of bare metal exposed to bromine.

#### Comparison of PTFE tape to bulk PTFE

A PTFE-coated stir bar was cut apart, the inside magnet was removed, and any contaminants were removed by immersion in concentrated hydrochloric acid. Bromine (0.30 mL) was added to the hollow PTFE shell and its top closed with a rubber septum. The bromine-filled shell was then immersed in dichloromethane. In an alternative experiment, PTFE tubing was filled with bromine and run through a two-necked flask containing dichloromethane, such that the PTFE portion of the tubing was immersed in the solvent and both ends of the tubing were outside of the sealed flask.

#### Effect of a PTFE stirring bar on bromination of benzene

A stock solution of 15.0 mL of benzene, 0.50 mL of bromine, and 0.50 mL of *n*-pentadecane (internal standard) was prepared in the dark. Seven 4 mL vials were prepared and 2.1 mL of the stock solution was added to each vial under a red light. Vial 1 was clear and the remaining were amber. Vials 1 and 2 were control experiments while stirring bars were added to the vials 3–7. The stirring bars were: 3) cut so that the iron bar was exposed, 4) unused small (0.2 cm), 5) unused large (0.4 cm), 6) used large, and 7) used small. All reactions were carried out in the dark. Periodically, samples were taken from each vial under a red light and analyzed by means of GC–MS.

## Supporting Information

File 1 Additional results and experimental details.
