# Supplementary material for: Properties of PTFE tape as a semipermeable membrane in fluorous reactions
Source: Beilstein J Org Chem. 2015 Jun 9;11:980–93. doi: 10.3762/bjoc.11.110 (PMC4505189; doi:10.3762/bjoc.11.110)
Supplement: File 1 — Additional results and experimental details. [file Beilstein_J_Org_Chem-11-980-s001.pdf]

Supporting Information

for

Properties of PTFE tape as a semipermeable membrane in fluorous reactions

Brendon A. Parsons[1,2], Olivia Lin Smith[1], Myeong Chae[1] and Veljko Dragojlovic[1]*

Address: [1]Wilkes Honors College of Florida Atlantic University, 5353 Parkside Drive, Jupiter, FL 33458, USA and [2]current address: Department of Chemistry, University of Washington, Box 351700, Seattle, WA 98195-1700, USA

Email: Veljko Dragojlovic - vdragojl@fau.edu

* Corresponding author

Additional results and experimental details

Experimental procedures and additional experimental details

Chemicals and supplies

All reagents (bromine, cyclohexene, 30% aqueous hydrogen peroxide, rubrene, 9,10-bis(phenylethynyl)anthracene, bis(2,4-dinitrophenyl)oxalate and bis(2-carbopentyloxy-3,5,6-trichloropheny)oxalate) and solvents (benzene, dichloromethane, ethyl acetate, hexane, dimethyl phthalate, acetonitrile, and *tert*-butanol) were obtained from Sigma-Aldrich or Fisher Scientific and were used as purchased. Dimethyl ester **1** was prepared according to a previously published procedure [1]. Taega Technologies High Density PTFE tape (1-inch width) was purchased from Fisher Scientific (Cat. No. 14610121). The PTFE syringe tube was purchased from Aldrich (gauge 16, 12 in long, cat. no. Z126772).

Construction of delivery tubes

A delivery tube was made of a glass tube which was sealed on each end with a PTFE tape. In the preparation of a delivery tube, one end of the tube was first covered with the PTFE tape; this was the end of the tube which was inserted into the substrate-containing vessel. The reagent was then added to the tube, and the other end was sealed with PTFE tape to reduce any evaporative loss. Care must be taken to stretch the PTFE over the end of the tube in only the lengthwise direction of the tape. PTFE tape is highly stretchable in the widthwise direction and such stretching reduces the thickness of the tape, which may alter the rate at which reagent diffuse across it. A variety of glass tubes are suitable for construction of delivery tubes. It was most economical to construct the delivery tubes from disposable glass Pasteur pipettes. Van Zee and Dragojlovic provide an illustrated guide to the construction of delivery tubes in their Supporting Information section [2].

Solvent transport in the course of a simple PV-PTFE diffusion of bromine into solvent

Experimental. The 10 mL round bottom flask and delivery tube set-up (Figure S1) was placed next to a ruler within the camera frame. A Canon PowerShot S500 Digital Elph was set to take pictures on a time interval using Canon RemoteCapture software. The images were recorded in macro mode with auto-focus and flash enabled and ISO set to automatic. The maximum column height reached by the bromine solution was quantified by measuring the number of vertical pixels in the column and correlating this number to pixel length of the ruler. Additionally, a stopwatch was also placed in-frame so that the time to reach the maximum height was known. In each experiment, 0.10 mL (0.20 mmol) of bromine was placed into the delivery tube, which was sealed on both ends with PTFE tape. The round bottom flask was filled with 5.0 mL of organic solvent. The delivery tube was inserted into the flask so that the lower tip of the delivery tube was immersed in the solvent. This process was repeated in triplicate with each of three solvents: dichloromethane, ethyl acetate, and hexanes.

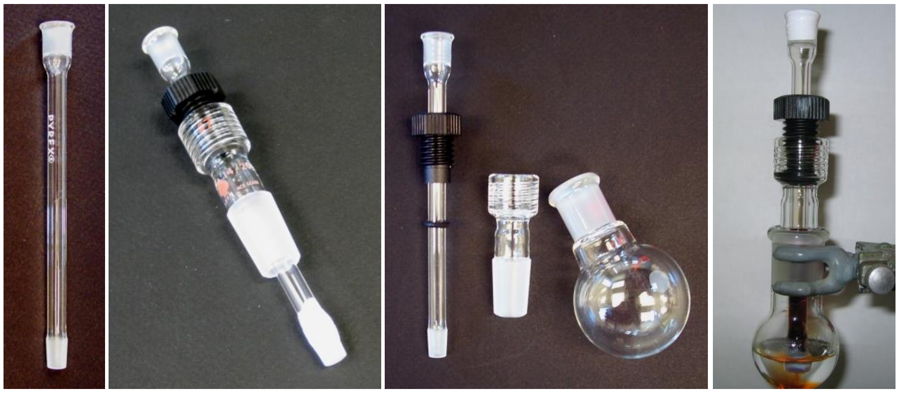

Figure S1: Set-up for PV-PTFE delivery of bromine into solvent.

Solvent transport in the course of PV-PTFE bromination of cyclohexene

Experimental. Cyclohexene (0.20 mmol) was dissolved in 5.0 mL of solvent (dichloromethane, ethyl acetate, and hexanes) in a 10 mL round bottom flask. Using the same set-up as in the previous experiment, 0.20 mmol of bromine was delivered from a PTFE-tipped delivery tube to react with the cyclohexene dissolved in the solvent. This reaction was repeated in triplicate with dichloromethane and ethyl acetate.

Additional information about Figure 8 in the manuscript. Figure 8 in the manuscript shows the iodolactonization of the unsaturated diester **1** shown below.

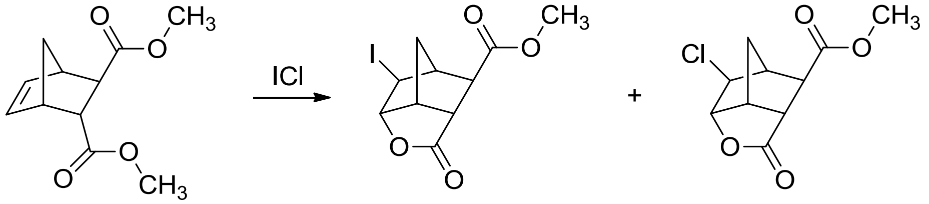

1 **2** **3**

Iodolactone **2** was obtained in 93–99% yield in various trials. The conversion of the starting materials was complete and the product was pure. No other byproducts, including chlorolactone **3**, were observed. Work up consisted of treatment with aqueous thiosulfate and filtration of the crude product through a short plug of silica gel. Iodolactone **2** is a known compound and we reported its analytical data in an earlier publication [3].

Solvent-assisted transport through PTFE tape

Experimental. A small amount of methyl red was dissolved in dimethyl phthalate (10.0 mL). Several delivery tubes were prepared by cutting off the tips of glass pipettes, capping the flat tops with PTFE tape, then inverting the pipettes so that they could be filled with the dyed phthalate. The prepared delivery tube were secured into 4 mL vial caps, then each was immersed in 2.0 mL of an organic solvent (dichloromethane, ethyl acetate, or hexanes) (Figure S2).

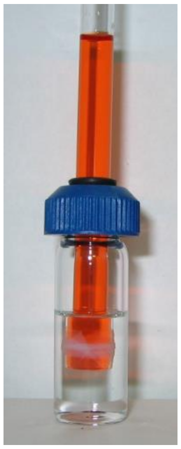

Figure S2: Set-up for dimethyl phthalate transport experiments.

Selective reagent transport in PV-PTFE chemiluminescence

Experimental. The set-up of this experiment was identical to that of the previous section testing the permeability to dimethyl phthalate. Two solutions were prepared for each reaction. An oxidizer solution of 10% hydrogen peroxide (H_2O_2) was prepared by diluting by volume 30% aqueous H_2O_2 with each of three solvents: water, acetonitrile, or *tert*-butanol. The second solution was 0.030 M solution of either bis(2-carbopentyloxy-3,5,6-trichlorophenyl)oxalate or bis(2,4-dinitrophenyl)oxalate and 0.0025 M rubrene in dimethyl phthalate. The reactions were performed in two different experimental set ups. The first reaction was set up with the peroxide solution in the delivery tube and the oxalate–rubrene solution in the vial (Figure S3a). In the second set up, the oxalate–rubrene solution was placed in the delivery tube with the peroxide solution in the vial (Figure S3b). Observations were made by means of time interval photography, using the same equipment as in previous experiments. The images were recorded in macro mode with auto-focus enabled, flash disabled, and ISO set to 400.

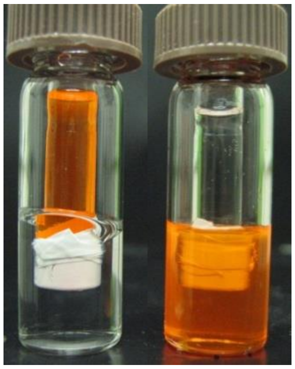

(a) (b)

Figure S3: Set-up for chemiluminescence experiments.

Additional information about Figures 11–14 from the manuscript. The light generated by the reactions in Figures 11a, 11b and 12 is orange in color due to the dyeing effect of rubrene in relatively high (0.0025 M) concentration. The concentration of rubrene was reduced to 0.00041 M for the Figures 11c, 11d, 13 and 14. Following this change, the chemiluminescence reactions gave off light of the expected yellow color. In Figure 14, 0.0020 M solution of 9,10-bis(phenylethynyl)anthracene was used. A photograph alternative to Figure 14, in which rubrene was used, was published in our earlier review article [4].

Comparison of PTFE tape to bulk PTFE

Effect of a PTFE stirring bar on bromination of benzene. Only bromobenzene was observed as a reaction product. Use of a large excess of benzene prevented possible problems with di- and tribromination. Bromination of pentadecane was not observed.

The experiment suffered from numerous reproducibility problems and was rather difficult to remove all the external factors. All the reactions were carried out in the dark. Initially, rubber septa were used to seal the reaction vials. However, septa first swell, then hardened and finally cracked releasing some fumes out of the reaction mixtures. We replaced septa with plastic (Bakelite) caps. That in turn meant that we had to open each vial to take a sample for analysis. As we did so, some hydrogen bromide, generated in the course of cut stirring bar reactions, entered other vials and catalyzed bromination of benzene. At the end, only the vials in which the same experiment was carried out were kept in the same dark cupboard. That ensured reasonably reproducible reaction conditions. Still, about 25–30% of the experiments were outliers and bromobenzene was produced in higher amounts. Results presented in the manuscript are representative examples, excluding the outliers.

References

1. Windmon, N.; Dragojlovic, V. *Green Chemistry Letters and Reviews*, **2008**, *1*, 155-163.
2. Van Zee, N. J.; Dragojlovic. V. *Org. Lett.* **2009**, *11*, 3190-3193.
3. Windmon, N.; Dragojlovic, V. *Beilstein J. Org. Chem.* **2008**, *4*, No. 29.
4. Van Zee, N. J.; Dragojlovic. V. *Chem. Eur. J.* **2010**, *16*, 7950-7958, Figure 13c.
